# Recent advances in understanding bile acid homeostasis

**DOI:** 10.12688/f1000research.12449.1

**Published:** 2017-11-20

**Authors:** John YL Chiang

**Affiliations:** 1 Department of Integrative Medical Sciences, Northeast Ohio Medical University, Rootstown, USA

**Keywords:** Bile acid synthesis, metabolic regulation, gut microbiota

## Abstract

Bile acids are derived from cholesterol to facilitate intestinal nutrient absorption and biliary secretion of cholesterol. Recent studies have identified bile acids as signaling molecules that activate nuclear farnesoid X receptor (FXR) and membrane G protein-coupled bile acid receptor-1 (Gpbar-1, also known as TGR5) to maintain metabolic homeostasis and protect liver and other tissues and cells from bile acid toxicity. Bile acid homeostasis is regulated by a complex mechanism of feedback and feedforward regulation that is not completely understood. This review will cover recent advances in bile acid signaling and emerging concepts about the classic and alternative bile acid synthesis pathway, bile acid composition and bile acid pool size, and intestinal bile acid signaling and gut microbiome in regulation of bile acid homeostasis.

## Introduction

Bile acid synthesis is tightly regulated by a network of feedback mechanisms that is complex and not completely understood. Alteration of bile acid homeostasis affects hepatic metabolic homeostasis and causes hepatic inflammation and pathogenesis of metabolic diseases such as non-alcoholic fatty liver disease (NAFLD), diabetes, and inflammatory bowel diseases. Recent research using mouse genetic models and human patients has shown that bile acids are signaling molecules that activate nuclear farnesoid X receptor (FXR), membrane G protein-coupled bile acid receptor-1 (Gpbar-1, also known as Takeda G protein-coupled receptor 5, or TGR5), and sphingosine-1-phosphate receptor 2 (S1PR2) to regulate not only bile acid synthesis in the liver but also lipid, glucose, and energy metabolism in tissues, including the liver, intestine, macrophages, and adipose tissues. Many investigators are not familiar with the concepts of bile acid homeostasis or bile acid pool and composition in maintaining metabolic homeostasis. This review will briefly discuss recent advances in understanding how bile acid homeostasis is maintained by (1) the classic bile acid synthesis pathway versus the alternative bile acid synthesis pathway, (2) regulation of bile acid pool size versus composition, (3) FXR signaling in liver versus intestine, and (4) the gut microbiota-to-liver axis. Only key references published in last 3–4 years are cited.

## Bile acid synthesis

**Figure 1.**
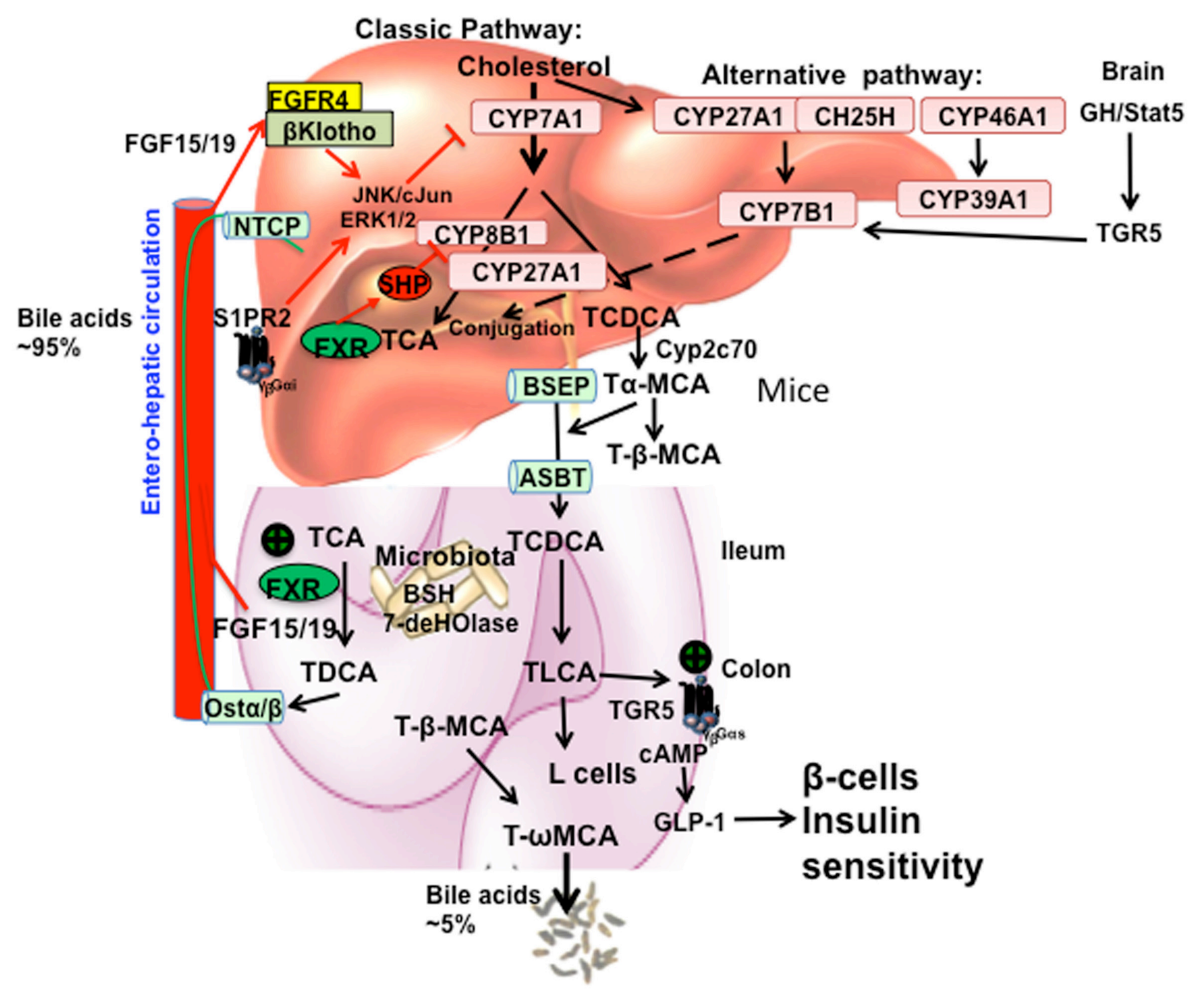
Bile acid synthesis, signaling, and regulation in human liver. In the liver, the classic bile acid synthesis pathway is initiated by cholesterol 7α-hydroxylase (CYP7A1) to synthesize cholic acid (CA), which requires sterol 12α-hydroxylase (CYP8B1), and chenodeoxycholic acid (CDCA). Mitochondrial sterol 27-hydroxylase (CYP27A1) oxidizes the steroid side chain, followed by peroxisomal β-oxidation to cleave a 3-C unit to form C24-bile acids. The alternative bile acid synthesis pathway is initiated by CYP27A1, followed by a non-specific oxysterol 7α-hydroxylase (CYP7B1) to synthesize both CA and CDCA in hepatocytes. CYP27A1 is also expressed in macrophages and many extrahepatic tissues for synthesis of steroid intermediates, which can be used for synthesis of bile acids in hepatocytes. In the brain, cholesterol is oxidized to 24-hydroxycholesterol by sterol 24-hydroxylase (CYP46A1), followed by a brain-specific sterol 7α-hydroxylase (CYP39A1). Cholesterol also can be hydroxylated to 25-hydroxycholesterol by cholesterol 25-hydroxylase (CH25H). These oxidized steroid intermediates formed in extrahepatic tissues can be transported to hepatocytes for synthesis of bile acids. In the brain, growth hormone-Stat5 signaling may activate G protein–coupled bile acid receptor-1 (Gpbar-1, also known as TGR5) to regulate expression of CYP7B1, a male-predominant enzyme. In mice, CDCA is converted to 6β-hydroxylated bile acids, α-muricholic acid (MCA) and β-MCA by Cyp2c70. Bile acids are conjugated to the amino acids taurine or glycine for secretion into bile via bile salt export pump (BSEP). In the ileum, TCA and TDCA are taken up into enterocytes via apical bile salt transporter (ASBT). In the colon, bile acids are de-conjugated by bacterial bile salt hydrolase (BSH) and are 7α-dehydroxylated by bacterial 7α-dehydroxylase to form deoxycholic acid (DCA) and lithocholic acid (LCA). Conjugated bile acids are secreted into portal blood via organic solute transporter α/β (OSTα/OSTβ) and circulated back to hepatocytes via Na-taurocholate co-transport peptide (NTCP) to inhibit bile acid synthesis. The enterohepatic circulation of bile acids is very efficient; approximately 95% bile acids are recovered and the approximately 5% bile acids that are lost in feces are replenished by *de novo* bile acid synthesis. Two mechanisms have been proposed to inhibit *CYP7A1* and *CYP8B1* gene transcription. In the liver, CDCA activates FXR to induce small heterodimer partner (SHP), which represses trans-activation of the *CYP7A1* and *CYP8B1* genes. In the intestine, CDCA activates FXR to induce fibroblast growth factor 15 (FGF15, or human orthologue FGF19), which is circulated to the liver to activate hepatic FGF receptor 4/Klotho signaling to inhibit *CYP7A1*/ *CYP8B1* gene transcription via ERK1/2/cJun of the mitogen-activated protein kinase (MAPK) pathway. In the liver, TCA-activated sphingosine-1-phosphate receptor 2 (S1PR2) signaling may activate the ERK1/2 pathway to modulate CYP7A1/CYP8B1 activity. In the intestinal L cells, TLCA activates TGR5 to increase cAMP and stimulate GLP-1 secretion. GLP-1 stimulates insulin secretion from β cells to improve insulin sensitivity.

Bile acid synthesis involves about a dozen enzymes located in the cytosol, endoplasmic reticulum, mitochondria, and peroxisomes to convert cholesterol to bile acids in hepatocytes ^1^. There are two major pathways, the classic (or neutral) pathway and the alternative (or acidic, sterol 27-hydroxylase [CYP27A1]) pathway in the liver ( Figure 1). The classic pathway starts with modifications of the steroid rings by hydroxylation, isomerization, and reduction/dehydroxylation enzymes located in cytosol and endoplasmic reticulum, followed by steroid side-chain oxidation in mitochondria and oxidative cleavage of the side chain in peroxisomes. The alternative pathway starts with oxidation of the steroid side chain and followed by 7α hydroxylation of the steroid ring to form oxidized steroid intermediates.

The classic pathway of bile acid synthesis is initiated by the rate-limiting enzyme cholesterol 7α-hydroxylase (CYP7A1) to form 7α-hydroxycholesterol, which is converted to 7α-hydroxy-4-cholesten-3-one (named C4). C4 is the common precursor of cholic acid (CA) and chenodeoxycholic acid (CDCA). Serum C4 level is now used as a biomarker for the rate of bile acid synthesis. C4 can be used for CA synthesis involving sterol 12α-hydroxylase (CYP8B1). Without 12α-hydroxylation, C4 is converted to CDCA. Mitochondrial CYP27A1 catalyzes the steroid side-chain oxidation, followed by peroxisomal β-oxidation to cleave a 3-C propionyl group from the side chain to form C24-bile acids ( Figure 1). The alternative pathway is initiated by CYP27A1 and is followed by a non-specific oxysterol 7α-hydroxylase (CYP7B1) in the liver, steroidogenic tissues, and macrophages. In the brain, cholesterol is converted to 24-hydroxycholesterol by sterol 24-hydroxylase (CYP46A1), followed by a brain-specific cholesterol 7α-hydroxylase (CYP39A1). In mouse and human liver, cholesterol 25-hydroxylase (CH25H, not a CYP enzyme) converts cholesterol to 25-hydroxycholesterol, which is the most abundant oxysterol in serum and can be used for synthesis bile acids in the liver. There is a misconception that the alternative pathway synthesizes CDCA only. However, the oxysterol intermediates formed in the extrahepatic tissues can be transported to the liver for synthesis of both CDCA (~70%) and CA (~30%). Many investigators assay CYP27A1 expression as a marker for the alternative pathway. It should be noted that CYP27A1 is required for bile acid synthesis in both classic and alternative pathways. CYP27A1 is not a rate-limiting enzyme in the alternative pathway, because CYP27A1 is highly expressed in most tissues. CYP7B1 is a marker for the alternative pathway. In mouse liver, CDCA is converted to α-muricholic acid (α-MCA) by a newly identified sterol 6β-hydroxylase (Cyp2c70). The 7α-OH group in α-MCA is epimerized to 7β-OH to form β-MCA ^2^. The conjugated bile acids are secreted into bile via the canalicular bile salt export pump to form mixed micelles with cholesterol and phosphatidylcholine in the canalicular and stored in the gallbladder. Meal intake stimulates cholecystokinin secretion from the pancreas to stimulate gallbladder contraction to release bile acids into the gastrointestinal tract. Some bile acids are passively absorbed in the upper intestine, but most are actively absorbed via apical sodium-dependent bile salt transport peptide in the ileum and colon where gut microbial bile salt hydrolase (BSH) de-conjugates bile acids to free bile acids, then bacterial 7α-dehydroxylase converts CA and CDCA to deoxycholic acid (DCA) and lithocholic acid (LCA), respectively. Some free bile acids (DCA and CA) may cross the colonic epithelium by passive diffusion. LCA is sulfur-conjugated and excreted into feces. A small amount of LCA circulated to the liver is rapidly sulfur-conjugated by bile salt sulfotransferases (SULTs), and excreted into urine. In mouse liver, LCA can be converted to ursodeoxycholic acid (UDCA) by 7β-hydroxylase as a primary bile acid. Human liver synthesizes very little UDCA (~1%).

### Enterohepatic circulation of bile acids

In the enterocytes, bile acids bind to ileum bile acid binding protein and trans-cross to the sinusoidal membrane, where organic solute transporter α (Ostα) and Ostβ heterodimer excreted bile acids to portal blood for circulation back to hepatocytes. Enterohepatic circulation of bile acids is a highly efficient physiological pathway, which recovers about 95% of bile acids in the pool and serves as a feedback mechanism to inhibit *CYP7A1* gene transcription and bile acid synthesis and maintain bile acid homeostasis in humans. In rats, enterohepatic circulation of bile acids is less efficient than in humans and the bile acid pool size is larger, and this may be due to absence of the gallbladder and relatively long intestine for the body size and higher bile acid synthesis rate. The Cyp7a1-specific activity in rat liver is about 100-fold higher than in human and mouse liver.

## Bile acid homeostasis

### 1. The classic bile acid synthesis pathways versus the alternative bile acid pathway

CYP7A1 is the only rate-limiting enzyme in the classic bile acid synthesis pathway. CYP8B1 is at the branch point for CA and CDCA synthesis and determines the ratio of 12α-hydroxylated bile acids (CA and DCA) to non-12α-hydroxylated bile acids (CDCA and LCA) in the bile acid pool. CA has the lowest critical micellar concentration (~50 μM) among all bile acids and is highly efficient for mixed micelle formation with cholesterol and phosphatidylcholine in bile and for absorption of dietary cholesterol in enterocytes. For that reason, CA is added in a high-cholesterol diet (lithogenic diet, 0.2% cholesterol and 0.5% CA) to accelerate hypercholesterolemia in rodents. It has been reported that the increased ratio of serum 12α-hydroxylated bile acids to non-12α-hydroxylated bile acids is associated with insulin resistance in humans ^3^. The alternative (acidic) pathway is the major pathway for bile acid synthesis in the neonate. After weaning, CYP7A1 is expressed and the classic pathway becomes the major pathway for bile acid synthesis in adult liver. The classic pathway is the predominant pathway for synthesis of bile acids in human liver, whereas the classic and alternative pathways contribute about equally to bile acid synthesis in rodents. In patients with bile acid synthesis deficiency in the classic pathway, the alternative pathway is used to produce bile acids. In *Cyp7a1 ^−/−^* mice, the alternative pathway is stimulated to produce bile acids to maintain a smaller but more hydrophilic bile acid pool with reduced tauro-cholic acid (TCA) and increased T-MCAs and taurodeoxycholic acid ^4^.

### 2. Bile acid pool size versus bile acid composition

Bile acid pool size: The bile acid pool is defined as the total bile acids circulating in the enterohepatic circulation, including bile acids in the liver (~1–2 %), intestine (~75–80%), and gallbladder (~15–20%). Small amounts (~1%) of bile acids spilled over from the circulation to serum and urine are not included in the pool. During cholestatic liver injury, serum bile acids are increased and cause jaundice. It is therapeutically important to assay the total bile acid pool size and changes in bile acid contents in the liver, intestine, and gallbladder bile.

Bile acid composition: Most bile acids in the pool are conjugated bile acids. Bile acid composition is different in the liver, gallbladder, intestine (ileum, cecum, and colon), feces, and serum. The gallbladder bile acid composition (bile) more closely represents the bile acids in the pool, including bile acids synthesized in the liver and circulated from enterohepatic circulation. Most studies report bile acid composition in serum, which does not represent bile acid composition in the pool. Whenever possible, bile acid composition in bile or liver should be determined to represent bile acid composition in the pool. In humans, bile acid pool consists of CA (~40%), CDCA (~40%), and DCA (~20%), the ratio of glycine- to taurine-conjugated bile acids is about 3 to 1, and the pool is highly hydrophobic. In mice, most bile acids are taurine-conjugated, the bile acid pool consists of CA (~60%) and α-MCA and β-MCA (~40%), and the pool is highly hydrophilic. Increased bile acid hydrophobicity index has been linked to cholesterol gallstone formation.

Feeding hydrophilic bile acids (such as T-MCA) inhibits intestinal cholesterol absorption, while feeding CA increases cholesterol absorption. Ablation of *Cyp8b1* ( *Cyp8b1 ^−/−^*) prevents atherosclerosis in *Apoe ^−/−^* mice. *Cyp8b1 ^−/−^* mice have improved glucose homeostasis by increased GLP-1 secretion ^5^. Germ-free mice, *Cyp8b1 ^−/−^* mice, and antibiotic-treated mice share common phenotypes; that is, all have increased CYP7A1 expression and enlarged bile acid pool with reduced TCA and increased T-MCA compared to wild type mice, and these mice are resistant from diet-induced obesity (DIO) ^6^. In transgenic mice overexpressing Cyp7a1 ( *Cyp7a-Tg*), increasing bile acid synthesis stimulates *de novo* cholesterol synthesis, biliary bile acid and cholesterol secretion, and fecal excretion to maintain bile acid and cholesterol homeostasis and prevent DIO and diabetes ^7^. Bile acid sequestrants reduce bile acid pool size, induce bile acid synthesis, and reduce intracellular cholesterol. As a consequence, *de novo* cholesterol synthesis, as well as low-density lipoprotein (LDL) receptor-mediated uptake of LDL cholesterol, is stimulated to reduce hypercholesterolemia. On the other hand, *Cyp7a1 ^−/−^* mice have a reduced bile acid pool size but surprisingly, also have improved glucose tolerance and insulin sensitivity ^4^. In *Cyp7a1 ^−/−^* mice, bile acid synthesis is switched to the alternative pathway to produce less TCA but more T-αMCA and T-βMCA, which antagonizes intestinal FXR activity to reduce ceramide synthesis and increase insulin sensitivity. Thus, bile acid composition, rather than bile acid pool size, plays an important role in regulation of bile acid and cholesterol homeostasis and protects against DIO and insulin resistance.

## Bile acid signaling in metabolic regulation

Emerging research in the last two decades has uncovered that bile acids are endogenous signaling molecules that activate the nuclear receptor FXR and membrane (TGR5, also known as Gpbar-1) and S1PR2 in the gastrointestinal system ^7^. Extensive experiments in mice and humans have demonstrated that FXR and TGR5 play critical roles in the regulation of bile acid synthesis and homeostasis ( Figure 1). However, the physiological role of these signaling pathways under normal physiological conditions is still not clear and not completely understood. Also, most studies were performed in mice, which have a very different bile acid composition and pool size compared with humans.

### FXR signaling

FXR signaling has been shown to play important roles in the regulation of metabolism in the liver, intestine, and adipose tissues. In the liver, CDCA (EC _50_, effective concentration that gives half maximal response, = ~17 μM) activates FXR to induce the negative nuclear receptor small heterodimer partner (SHP) to inhibit transcription of the *CYP7A1* and *CYP8B1* genes ( Figure 1). In mice, CA is the predominant bile acid in the bile acid pool but is a very weak FXR agonist (EC _50_ = ~0.59 mM). It is not likely that the hepatic TCA concentration (~100 μM) is high enough to activate FXR under normal physiological conditions. The FXR/SHP mechanism may be activated when bile acids are accumulated at high levels in hepatocytes under cholestatic conditions to inhibit bile acid synthesis as an adaptive response to liver injury. The intestinal FXR/FGF15/hepatic FGF receptor 4/β-Klotho pathway may activate JNK/ERK of the mitogen-activated protein kinase (MAPK) pathway to inhibit *CYP7A1* and *CYP8B1* gene transcription ( Figure 1). This may be a more physiologically relevant pathway mediating bile acid feedback via enterohepatic circulation to regulate bile acid synthesis.

### TGR5 signaling

Secondary bile acids (LCA, EC _50_ = ~0.03 μM; DCA, EC _50_ = ~1 μM) produced in the intestine (colon) by gut bacteria activate TGR5 signaling, which induces cAMP/PKA signaling to stimulate energy metabolism in brown adipose tissue, relax and refill the gallbladder, and secrete glucagon-like peptide 1 (GLP-1) from intestinal endocrine L cells ( Figure 1) ^8^. A recent study reports that TGR5 may be involved in the regulation of CYP7B1, a sexually dimorphic and male-predominant gene in the alternative bile acid synthesis pathway ^9^. Activation of TGR5 in macrophages and Kupffer cells inhibits pro-inflammatory cytokine production and cholangiocyte proliferation ^10^.

### S1PR2 signaling

S1PR2 is a TCA-activated G _αi_ protein-coupled receptor, which activates ERK1/2 and AKT in hepatocytes and cholangiocytes ^11^. It has been shown that bile acids activate ERK1/2 and JNK/cJun to inhibit *Cyp7a1* and *Cyp8b1* gene transcription ( Figure 1). However, the role of the S1PR2 pathway in the regulation of bile acid synthesis is not clear and requires further study.

## The gut microbiota and bile acid homeostasis

The gut-to-liver axis plays a critical role in bile acid metabolism. Bile acids control gut bacteria overgrowth and the gut microbiota regulates bile acids synthesis, bile acid pool size and composition, and enterohepatic circulation of bile acids. Bile acids reshape the gut microbiome, which has great impact on host metabolism and metabolic diseases ^12– 15^. Activation of FXR by specific agonists inhibits bile acid and fatty acid synthesis and improves glucose and insulin sensitivity in obese and diabetic mice. However, reported results are not consistent and are controversial ^7^. In general, activation of liver FXR signaling is beneficial for primary biliary cirrhosis and non-alcoholic steatohepatitis (NASH) by reducing bile acid pool and progression to fibrosis ^16, 17^.

The role of intestinal FXR signaling in metabolic regulation is controversial. Deficiency of intestinal FXR or antagonizing FXR by an antioxidant, Tempol or Gly-MCA, an MCA derivative that is resistant to BSH activity, prevents DIO ^18, 19^. T-MCA has been shown to antagonize intestinal FXR activity and improve DIO and weight gain in mice and human patients ^20– 22^. Inhibition of intestinal FXR reduces ceramide synthesis and modulates hepatic gluconeogenesis in mice ^23^. In contrast, the intestine-restricted FXR agonist fexaramine reduces weight and insulin resistance and may occur via FGF15-mediated adipocyte browning ^24^. Activation of intestinal FXR and TGR5 crosstalk in intestinal L cells stimulates TGR5-mediated GLP-1 secretion to improve insulin sensitivity ^8^. Activation of FXR by GW4064 in intestinal L cells was shown to decrease proglucagon expression by interfering with glucose-responsive carbohydrate-response element binding protein and GLP-1 secretion ^25^. Bile acids are known to stimulate glucose-induced GLP-1 secretion. It is possible that mice housed in different facilities, their genetic background, and differential effects of FXR agonists on gut microbiota contribute to the observed paradoxical effects on glucose tolerance and insulin sensitivity.

## Future perspective

Bile acid signaling regulates hepatic metabolism via FXR signaling in the liver and intestine, and TGR5 in the intestine, to modulate the gut microbiome, host metabolism, and diseases. The emerging research in bile acid metabolism and homeostasis has been translated to drug therapy for liver diseases, NASH, and diabetes and obesity. Most bile acid research is conducted in mouse models, which have a very different bile acid composition and pool size. Gender differences in bile acids and microbiota in diet-induced steatosis have been reported and need to be further studied ^26, 27^. Bile acid receptors are signaling integrators and have emerged as therapeutic targets for the treatment of dyslipidemia, NAFLD, diabetes, and cardiovascular diseases ^17, 28^. Bariatric surgeries are effective in reducing weight and improving insulin resistance in obese patients, and FXR and TGR5 signaling have been implicated in increasing serum bile acids and FGF19 after bariatric surgery ^29– 31^. However, the underlying mechanism of bile acid signaling in improving diabetes after bariatric surgery is not clear and needs to be elucidated to develop therapeutic strategies to cure diabetes and NAFLD.

## Abbreviations

BSH, bile salt hydrolase; C4, 7α-hydroxy-4-cholesten-3-one; CA, cholic acid; CDCA, chenodeoxycholic acid; CYP7A1, cholesterol 7α-hydroxylase; CYP7B1, oxysterol 7α-hydroxylase; CYP8B1, sterol 12α-hydroxylase; CYP27A1, sterol 27-hydroxylase; DCA, deoxycholic acid; DIO, diet-induced obesity; EC _50_, concentration of a drug that gives half-maximal response; FGF15, fibroblast growth factor 15; FXR, farnesoid X receptor; GLP-1, glucagon-like peptide 1; Gpbar-1, G protein–coupled bile acid receptor-1; LCA, lithocholic acid; LDL, low-density lipoprotein; MCA, muricholic acid; NAFLD, non-alcoholic fatty liver disease; NASH, non-alcoholic steatohepatitis; Ost, organic solute transporter; S1PR2, sphingosine-1-phosphate receptor 2; SHP, small heterodimer partner; T-MCA, tauro-muricholic acid; TCA, tauro-cholic acid; TGR5, Takeda G protein receptor 5; UDCA, ursodeoxycholic acid.
